# Alkaline phosphatase predicts short-term postoperative outcome in adult patients with moyamoya disease

**DOI:** 10.3389/fneur.2025.1606348

**Published:** 2025-09-26

**Authors:** Wei Sun, Huihui Wang, Qiheng He, Junsheng Li, Chenglong Liu, Zhiyao Zheng, Siqi Mou, Bojian Zhang, Zhikang Zhao, Chuming Tao, Wei Liu, Xiangjun Shi, Yan Zhang, Peicong Ge, Dong Zhang, Jun Wu

**Affiliations:** ^1^Department of Neurosurgery, Beijing Tiantan Hospital, Capital Medical University, Beijing, China; ^2^Department of Hematology, The Second Xiangya Hospital, Central South University, Changsha, China; ^3^Research Unit of Accurate Diagnosis, Treatment, and Translational Medicine of Brain Tumors, Chinese Academy of Medical Sciences and Peking Union Medical College, Beijing, China; ^4^Medical School, University of Chinese Academy of Sciences, Beijing, China; ^5^China National Clinical Research Center for Neurological Diseases, Beijing, China; ^6^Department of Rheumatology and Immunology, Beijing Tiantan Hospital, Capital Medical University, Beijing, China; ^7^Department of Neurosurgery, Beijing Hospital, National Center of Gerontology, Beijing, China; ^8^Institute of Geriatric Medicine, Chinese Academy of Medical Sciences, Beijing, China

**Keywords:** alkaline phosphatase, moyamoya disease, biomarker, outcome, postoperative

## Abstract

**Background:**

Alkaline phosphatase (ALP) has played a pivotal role in vascular diseases in recent years. However, the association between ALP level and postoperative complications of moyamoya disease (MMD) has not been studied.

**Patients and methods:**

Blood samples were collected from recruited patients with MMD. The serum ALP concentrations of the patients were determined using non-frozen specimens via an automated enzymatic assay. Patients were then divided into two groups according to the median, and a comparative analysis was performed. Patients were stratified into two cohorts for statistical evaluation based on the occurrence of postoperative stroke events. We employed a nomogram to identify risk factors for postoperative events. We also created a Cox model to analyze the risk factors for postoperative stroke events, including ALP. Furthermore, we plotted a restricted cubic spline (RCS) of ALP concentration vs. postoperative stroke events.

**Results:**

We could find that the ALP concentration of non-postoperative stroke group and postoperative stroke group was 68.70 U/L and 71.15 U/L. This nomogram showed that ALP was a risk factor for postoperative events, and the Hosmer–Lemeshow goodness-of-fit test was employed, suggesting that the model was reliable (χ^2^ = 8.507, *p* = 0.386). And it also could obverse that there was a statistically positive correlation from the RCS between ALP concentration and postoperative stroke events Cox analysis (HR = 1.006, 95% CI = 1.002–1.010, *p* = 0.008).

**Conclusions:**

ALP levels may predict the short-term postoperative outcomes of MMD patients undergoing surgical treatment.

## Introduction

Moyamoya disease (MMD) is a rare cerebrovascular disease characterized by chronic stenosis or occlusion of the internal carotid arteries leading to the development of abnormal vascular networks near the base of the brain (1, 2). The etiology of MMD remains elusive, may involve a combination of genetic, immune, inflammatory, and environmental factors. However, the RNF213 gene has demonstrated a significant association with MMD. Research indicated that variations in the RNF213 gene, particularly the p.R4810K mutation, were closely linked to an increased risk of developing MMD in East Asian populations (3, 4). Its clinical presentation can range from ischemic stroke to hemorrhagic events, frequently resulting in neurological deficits (5). The current treatment options are primarily surgical, aimed at revascularization to improve cerebral perfusion (6), and perioperative medical decision-making and the selection of surgical modality are crucial for patient outcomes (7).

Despite advancements in surgical techniques, predicting short-term postoperative outcomes in patients with MMD remains a challenge. Clinicians have tried to stratify these patients based on clinical aspects, and recent studies suggest that several biomarkers and medical imaging technology may be helpful in predicting postoperative outcomes (8–12). However, the potential role of Alkaline Phosphatase (ALP) has not been elucidated.

ALP, a hydrolase enzyme responsible for dephosphorylating compounds, has been implicated in multiple metabolic processes including bone metabolism, liver function, and vascular calcification (13–16). While ALP has traditionally been associated with hepatic and biliary pathology, emerging evidence suggests that elevated ALP levels are associated with atherosclerosis and cardiovascular disease (17–20). Previous studies have demonstrated that serum ALP levels may serve as a prognostic biomarker for stroke, post-stroke recurrence, and postoperative stroke (19, 21–23). Currently, there is limited research on the association between ALP levels and clinical outcomes in patients with MMD.

In this study, we aimed to explore the predictive value of ALP levels in determining short-term postoperative outcomes in MMD patients undergoing revascularization surgery. Through retrospective analysis and prospective observation, we sought to elucidate whether preoperative ALP levels correlated with postoperative complications and overall functional recovery. By establishing a link between ALP and postoperative outcomes, we propose a novel biomarker that can aid in the prognostication of MMD and potentially guide therapeutic strategies for the postoperative management of patients with MMD.

## Patients and methods

### Study participants

**Figure 1:**
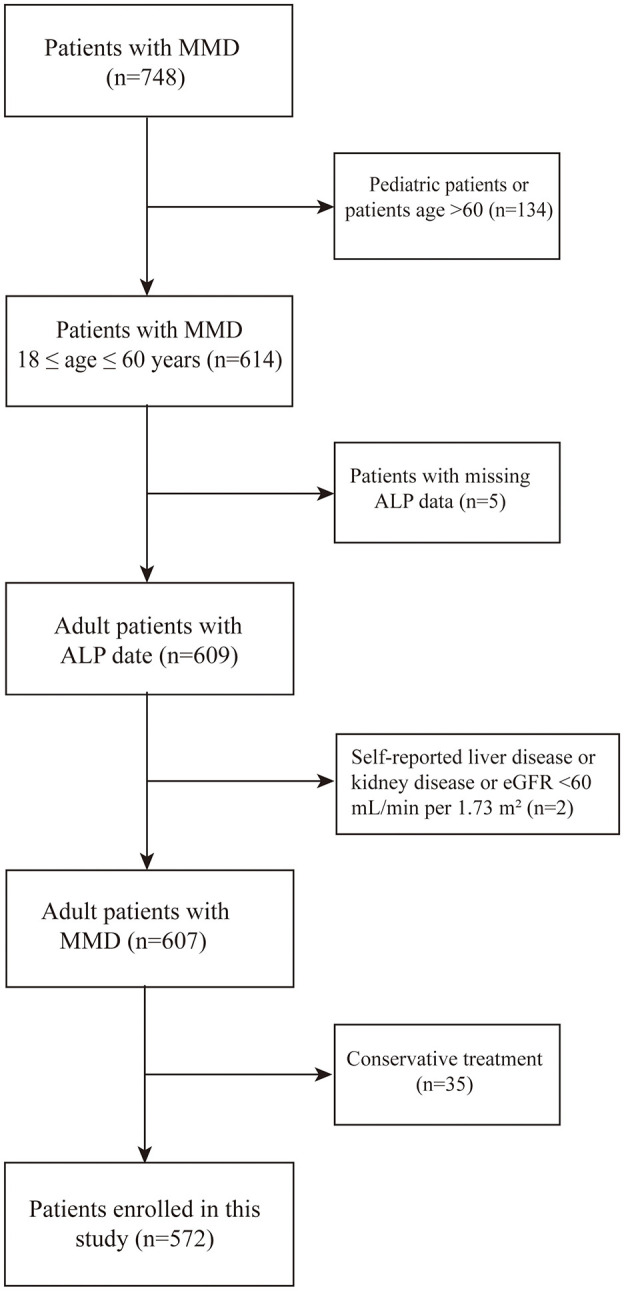
Flow diagram illustrating the participant screening procedure. HCs, healthy controls; MMD, moyamoya disease; ALP, Alkaline Phosphatase.

In this study, we enrolled 748 patients with MMD at the Department of Neurosurgery, Beijing Tiantan Hospital, Capital Medical University, from September 1, 2020, to December 31, 2021. The inclusion criteria were as follows: (1) patients that imaging findings revealed stenosis or occlusion of the internal carotid arteries and moyamoya vessel diagnosed as MMD according to the guidelines published in 2015 in Japan and 2023 AHA/ASA consensus statement (2) informed consent was provided (24). The exclusion criteria were as follows: (1) pediatric patients or patients aged over 60 years (*N* = 134); (2) inability to access postoperative clinical data (*N* = 5); (3) a history of hepatic or renal disorders or presented with an eGFR of < 60 ml/min (N = 2) (19); and (4) MMD patients who were not undergoing surgical treatment (*N* = 35) as presented in Figure 1. Ultimately, 572 patients were included in the analysis. The investigation was conducted in strict adherence to the principles outlined in the Declaration of Helsinki. Ethical approval for this research was granted by the Institutional Review Board of Beijing Tiantan Hospital, Capital Medical University (KY2022-051-02). Written informed consent were obtained from all participants.

### Data collection

Clinical data from individuals diagnosed with MMD were systematically aggregated and comprehensively analyzed. We collected patient demographic information, including age and gender, and recorded clinical characteristics including heart rate, blood pressure, and body mass index (BMI). The systolic blood pressure (SBP) and diastolic blood pressure (DBP) in patients with MMD were quantified using a conventional mercury sphygmomanometer. The cardiac rhythm was precisely quantified using electrocardiographic technology. BMI was determined using conventional methodology, which involves dividing the patient's weight (in kilograms) by the square of their height (in meters). Furthermore, we documented patient risk factors for cardiovascular and cerebrovascular diseases, such as smoking, alcohol consumption, hypertension, diabetes mellitus, hyperlipidemia, and the initial symptomatology of patients with MMD.

### Serum ALP concentration

It is imperative for patients to undergo a fasting period of at least 12 h prior to blood sample collection. Serum ALP concentrations were determined in non-frozen specimens using an automated enzymatic assay in the hospital. The normal range of our center is referred to 26–117 U/L. The quantification of ALP adhered to the guidelines recommended by the International Federation of Clinical Chemistry and Laboratory Medicine (IFCC) in 2011 (19).

### Treatment, follow-up and prognostic events

In general, there are three types of MMD surgery: direct bypass, indirect bypass and combined bypass (6). The surgical procedure was chosen based on the clinical guidelines for MMD treatment. Additionally, surgical intervention is prioritized for cerebral hemispheres with worse hemodynamics and more severe symptoms in clinical practice (25). Short-term outcomes were defined as postoperative complications that emerged within the one-month period after surgical interventions (26). After the patients were discharged, they were followed up in the outpatient department. Postoperative complications, including cerebral hemorrhage events, ischemic events, hydrocephalus, and subcutaneous effusion, were defined as all intracranial complications that occurred 1 month after surgery. Postoperative stroke events were defined as stroke that occurred after surgical treatment, including the ischemic subtype and hemorrhagic subtype (27). Initially, individuals with MMD were stratified into two distinct cohorts based on the median ALP concentration. Furthermore, patients were divided into two groups according to their prognostic outcomes: postoperative and non-postoperative stroke. ALP concentrations were used as predictive biomarkers for the incidence of perioperative complications following surgical interventions. The correlation between ALP concentrations and complications was demonstrated by restricted cubic splines (RCS).

### Statistical analysis

The R software (version 4.2.3, https://www.r-project.org) was used in this study. Categorical variables were represented as frequencies, whereas continuous variables were described using mean and standard deviation (SD) and median and interquartile range (IQR). Differences between categorical variables in the two groups were analyzed using Pearson's chi-square test and Fisher's exact test. Mann–Whitney *U* tests were used to examine the correlations between continuous and categorical variables.

In our study, both univariate and multivariate Cox regression analyses were performed to evaluate the risk factors associated with perioperative complications. We developed two models based on the Cox proportional hazards framework to investigate the association between serum ALP concentrations and the incidence of all perioperative complications and cerebral ischemic events. The crude model was concerned with the unadjusted model of serum ALP concentration. The multivariate model was developed through univariate analysis (*p* < 0.10), backward selection methods and considering carefully. Finally, we got a multivariate model, including age, gender, SBP, DBP, BMI, ALP level, and hemoglobin (HGB) concentration. Based on this multivariate model, a nomogram was constructed and evaluated using a calibration curve.

## Results

### Baseline disparities in MMD patients with differential levels of ALP

In this study, 572 patients diagnosed with MMD were included in the analysis. The patients were stratified into two cohorts according to ALP level based on the median value, and the baseline characteristics are presented in Table 1. In addition, patients with higher ALP levels had more smokers and were older than the low concentration group (all *p* < 0.05), and the age of the high and low concentration groups was 40.24 ± 9.40 and 41.88 ± 10.60. The lower group had more females, with 179 (62.6%) female individuals in the lower group and 141 (49.4%) female patients in the high-concentration group (*p* = 0.001). Patients with higher ALP levels exhibited increased white blood cell (WBC), Neutrophil, and RBC counts, and alanine transaminase (ALT), aspartate aminotransferase (AST), and HGB levels (all *p* < 0.05).

**Table 1:** Baseline characteristics with different levels of ALP.

**Variables**	**Comparison of baseline characteristics between groups of different ALP concentrations**			
	**All patients (*****n*** = **572)**	**Low group (*****n*** = **286)**	**High group (*****n*** = **286)**	***p*** **-value**
Age (year), mean ± SD	41.06 ± 10.04	40.24 ± 9.40	41.88 ±10.60	0.028^*^
Gender (female), *n* (%)	320 (55.9)	179 (62.6)	141 (49.4)	0.001^*^
**Clinical features, mean** ±**SD**				
Heart rate, bpm	78.83 ± 6.25	78.71 ± 6.16	78.95 ± 6.36	0.564
SBP, mmHg	131.91 ± 12.70	131.26 ± 12.61	132.56 ± 12.77	0.269
DBP, mmHg	82.20 ± 10.36	81.78 ± 13.00	82.63 ± 11.82	0.724
BMI, kg/m^2^	25.51 ± 4.20	25.15 ± 12.20	25.96 ± 22.91	0.099
**History of risk factors, (** ***n*** **%)**				
Current smoking	113 (19.8)	42 (14.7)	71 (24.8)	0.002^*^
Current alcohol abuse	69 (12.1)	29 (10.1)	40 (14.0)	0.158
Diabetes mellitus	81 (14.2)	35 (12.2)	46 (16.1)	0.187
Hypertension	211 (36.9)	98 (34.3)	113 (39.5)	0.194
Hyperlipidemia	73 (12.8)	34 (11.9)	39 (13.6)	0.531
**Laboratory results, median (IQR)**				
WBC count, 10^9^/L	6.81 (2.45)	6.60 (2.41)	7.05 (2.29)	0.006^*^
Lymphocyte count, 10^9^/L	1.92 (0.83)	1.89 (0.76)	1.97 (0.89)	0.277
Monocyte count, 10^9^/L	0.36 (0.16)	0.35 (0.17)	0.37 (0.15)	0.058
Neutrophil count, 10^9^/L	4.18 (1.89)	4.03 (1.90)	4.40 (1.88)	0.004^*^
SII	528.01 (363.35)	528.98 (354.14)	520.55 (377.98)	0.220
RBC count, 10^12^/L	4.64 (0.64)	4.55 (0.61)	4.76 (0.59)	< 0.001^*^
HGB, g/L	142.00 (23.00)	139.00 (20.00)	145.00 (24.00)	< 0.001^*^
PLT count, 10^9^/L	247.00 (77.00)	248.50 (79.00)	246.50 (74.00)	0.717
AST, U/L	17.50 (7.40)	16.90 (7.20)	18.15 (8.00)	0.001^*^
ALT, U/L	21.95 (18.10)	19.00 (15.50)	24.10 (18.80)	< 0.001^*^
**Surgical options, (** ***n*** **%)**				0.865
Indirect bypass	240 (42.0)	119 (41.6)	121 (42.3)	
Non-indirect bypass	332 (58.0)	167 (58.4)	165 (57.7)	

MMD, moyamoya disease; SD, standard deviation; SBP, systolic blood pressure; DBP, diastolic blood pressure; BMI, body mass index; IQR, interquartile range; WBC, white blood cell; RBC, red blood cell; HGB, hemoglobin; PLT, platelet; AST, aspartate aminotransferase; ALT, alanine transaminase; ALP, alkaline phosphatase.

^*^p < 0.05, significant difference.

### Comparative analysis of baseline characteristics among patients experiencing different postoperative events

In our study, patients were stratified into two cohorts according to their prognostic outcomes by conducting a comparative analysis. A total of 572 patients were divided into postoperative stroke (*N* = 100) and non-postoperative stroke (*N* = 472) groups as detailed in Table 2. We could find that the ALP concentration of non-postoperative stroke group and postoperative stroke group was 68.70 U/L and 71.15 U/L. It was observed that postoperative stroke patients had higher WBC, Neutrophil, and RBC counts and HGB and ALT levels than non-postoperative stroke patients (all *p* < 0.05). In the postoperative stroke group, individuals with a history of alcohol consumption, diabetes mellitus, and hypertension constituted a higher proportion of the cohort, and included a greater number of female patients (all *p* < 0.05). In addition, these patients had a lower heart rate (*p* = 0.017).

**Table 2:** Comparative analysis of baseline characteristics among patients experiencing different postoperative stroke events.

**Comparison of baseline characteristics between groups of different outcome**				
**Variables**	**All patients (*****n*** = **572)**	**Non-postoperative stroke (*****n*** = **472)**	**Postoperative stroke (*****n*** = **100)**	***p*** **-value**
Age (year), mean ± SD	41.06 ± 10.04	41.00 ± 10.04	41.39 ± 10.11	0.722
Gender (female), *n* (%)	320 (55.9)	275 (58.3)	45 (45)	0.015^*^
**Clinical features, mean** ±**SD**				
Heart rate, bpm	78.83 ± 6.25	79.08 ± 6.47	77.68 ± 4.96	0.017^*^
SBP, mmHg	131.91 ± 12.70	131.60 ± 13.12	133.36 ± 10.41	0.209
DBP, mmHg	82.20 ± 10.36	82.21 ± 10.65	82.18 ± 8.89	0.979
BMI, kg/m^2^	25.51 ± 4.20	25.45 ± 4.16	26.03 ± 4.41	0.210
**History of risk factors, (** ***n*** **%)**				
Current smoking	113 (19.8)	91 (19.3)	22 (22.0)	0.535
Current alcohol abuse	69 (12.1)	51 (10.8)	18 (18.0)	0.045^*^
Diabetes mellitus	81 (14.2)	59 (12.5)	22 (22.0)	0.013^*^
Hypertension	211 (36.9)	165 (35.0)	46 (46.0)	0.038^*^
Hyperlipidemia	73 (12.8)	59 (12.5)	14 (14.0)	0.683
**Laboratory results, median (IQR)**				
WBC count, 10^9^/L	6.81 (2.45)	6.72 (2.45)	7.22 (2.01)	0.006^*^
Lymphocyte count, 10^9^/L	1.92 (0.83)	1.90 (0.81)	2.08 (0.82)	0.055
Monocyte count, 10^9^/L	0.36 (0.16)	0.36 (0.16)	0.37 (0.17)	0.436
Neutrophil count, 10^9^/L	4.18 (1.89)	4.07 (1.87)	4.55 (1.79)	0.005^*^
SII	528.01 (363.35)	511.21 (371.01)	589.49 (301.24)	0.093
RBC count, 10^12^/L	4.64 (0.64)	4.61 (0.64)	4.82 (0.58)	0.006^*^
HGB, g/L	142.00 (23.00)	141.00 (24.00)	147.00 (21.00)	0.005^*^
PLT count, 10^9^/L	247.00 (77.00)	245.50 (78.00)	252.00 (68.00)	0.083
AST, U/L	17.50 (7.40)	17.55 (7.40)	17.00 (7.60)	0.759
ALT, U/L	21.95 (18.10)	21.20 (17.40)	24.25 (21.5)	0.015^*^
ALP, U/L	68.70 (25.70)	68.25 (26.10)	71.15 (27.00)	0.241
**Surgical options, (** ***n*** **%)**				0.073
Indirect bypass	240 (42.0)	190 (40.3)	50 (50.0)	
Non-indirect bypass	332 (58.0)	282 (282)	50 (50.0)	

MMD, moyamoya disease; SBP, systolic blood pressure; DBP, diastolic blood pressure; BMI, body mass index; WBC, white blood cell; RBC, red blood cell; HGB, hemoglobin; PLT, platelet; AST, aspartate aminotransferase; ALT, alanine transaminase; ALP, alkaline phosphatase.

^*^p < 0.05, significant difference.

**Figure 2:**
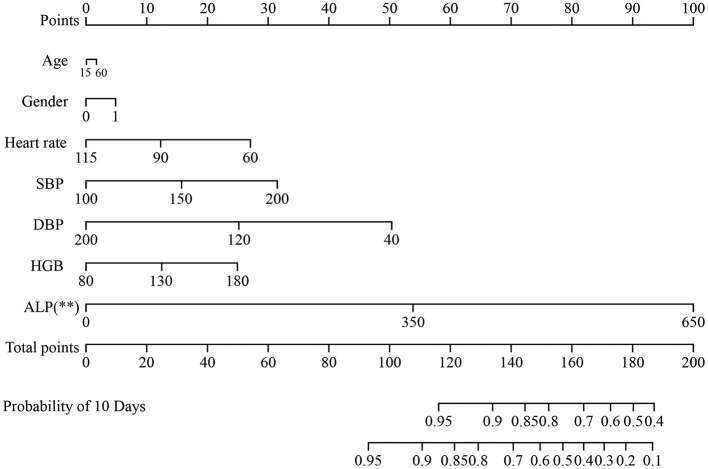
The nomogram for the prediction of outcome in patients with MMD undergoing surgical treatment. Age, gender, SBP, DBP, BMI, ALP and HGB concentrations. MMD were included. MMD, moyamoya disease; SBP, systolic blood pressure; DBP, diastolic blood pressure; BMI, body mass index; HGB, hemoglobin; ALP, alkaline phosphatase. **p < 0.01.

Furthermore, to identify risk factors for postoperative stroke, We plotted a nomogram (*p* = 0.004, 95% CI = 0.529-0.646, *c*-index = 0.600) based on this multivariate model (Figure 2). We also plotted the calibration curve (Supplementary Figure S1) of the nomogram to evaluate its predictive accuracy and the agreement between predicted and observed probabilities of cerebral ischemic events. The predictive accuracy of the model improved with longer follow-up periods, demonstrating progressively closer concordance between predicted and actual event rates over time.

### Cox analysis of risk factors for recurrence of postoperative stroke events in patients with MMD following surgical intervention

**Figure 3:**
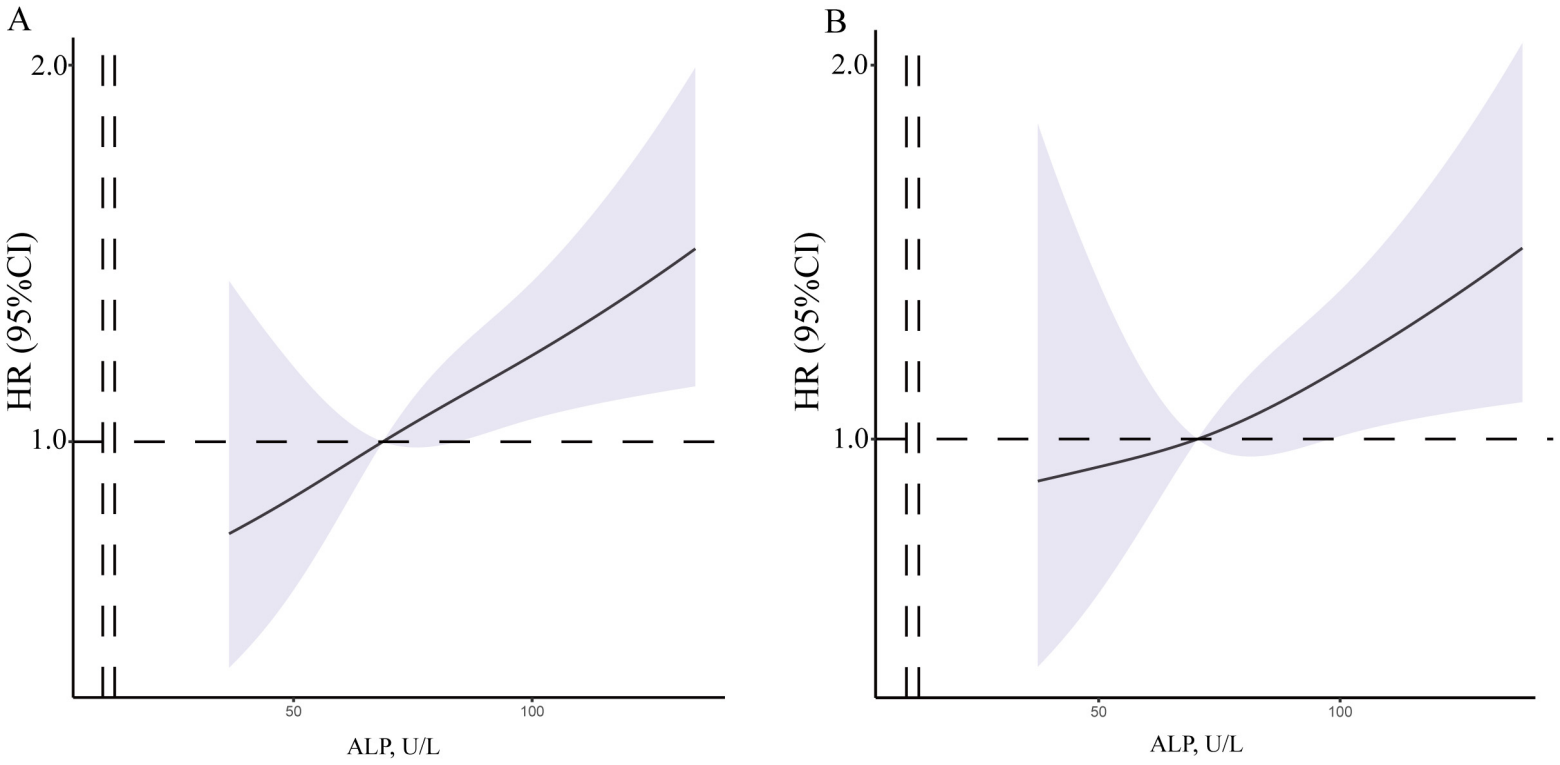
The RCS of between ALP concentration and postoperative stroke events. **(A)** The crude model was concerned the unadjusted model of serum ALP concentrations. **(B)** The final model was adjusted for age, gender, SBP, DBP, BMI, ALP and HGB concentrations. MMD, moyamoya disease; SBP, systolic blood pressure; DBP, diastolic blood pressure; BMI, body mass index; WBC, white blood cell; RBC, red blood cell; HGB, hemoglobin; PLT, platelet; AST, aspartate aminotransferase; ALT, alanine transaminase; ALP, alkaline phosphatase.

Univariate and multivariate Cox models were created to analyze the risk factors for postoperative stroke events (Table 3). In the univariate Cox model, we found that the level of ALP had positive correlated with postoperative stroke events (HR = 1.006, 95% CI = 1.002−1.010, *p* = 0.003, Figure 3A). Additionally, in the univariate Cox model, a negative correlation was identified between the incidence of postoperative stroke events in patients with MMD and heart rate (*p* = 0.043). There was a positive correlation between postoperative stroke events and gender, RBC count, HGB level, ALT level, and ALP (all *p* < 0.05). A significant correlation was also observed between ALP concentration and the incidence of postoperative stroke, as determined by the Cox analysis (HR = 1.006, 95% CI = 1.002–1.010, *p* = 0.008, Figure 3B), in the multivariable Cox model. Univariate and multivariate Cox models were used to analyze all perioperative risk factors. We also found that ALP level was positively associated with all perioperative complications in the univariate and multivariate Cox models (Supplementary Figure S2).

**Table 3:** Cox analysis of risk factors for postoperative stroke events in patients with MMD following surgical intervention.

**Variables**	**Univariable**			**Multivariable**		
	**HR**	**CI**	***p***	**HR**	**CI**	***p***
Age (year)	1.004	0.984–1.024	0.699	1.000	0.980–1.020	0.942
Gender (male), *n* (%)	1.608	1.084–2.384	0.018^*^	1.218	0.723–2.050	0.458
Heart rate, bpm	0.966	0.934–0.999	0.043^*^	0.981	0.949–1.014	0.253
SBP, mmHg	1.009	0.995–1.024	0.220	1.012	0.992–1.032	0.231
DBP, mmHg	0.999	0.980–1.018	0.925	0.987	0.963–1.012	0.305
BMI, kg/m^2^	1.028	0.984–1.074	0.216	1.009	0.963–1.059	0.700
Surgical options, (*n*%)	0.698	0.472–1.034	0.073			
WBC count, 10^9^/L	1.061	0.987–1.140	0.109			
Lymphocyte count, 10^9^/L	1.215	0.930–1.588	0.153			
Monocyte count, 10^9^/L	1.483	0.415–5.295	0.544			
Neutrophil count, 10^9^/L	1.048	0.967–1.135	0.256			
SII	1.000	1.000–1.000	0.746			
RBC count, 10^12^/L	1.544	1.033–2.308	0.034^*^			
HGB, g/L	1.015	1.003–1.027	0.011^*^	1.010	0.994–1.026	0.212
PLT count, 10^9^/L	1.002	0.999–1.005	0.144			
AST, U/L	1.010	0.992–1.028	0.277			
ALT, U/L	1.006	1.000–1.012	0.035^*^			
ALP, U/L	1.006	1.002–1.010	0.003^*^	1.006	1.002–1.010	0.008^*^

MMD, moyamoya disease; SBP, systolic blood pressure; DBP, diastolic blood pressure; BMI, body mass index; WBC, white blood cell; RBC, red blood cell; HGB, hemoglobin; PLT, platelet; AST, aspartate aminotransferase; ALT, alanine transaminase; ALP, alkaline phosphatase.

^*^p < 0.05, significant difference.

## Discussion

The role of biomarkers in predicting postoperative outcomes in patients with MMD is of interest. ALP has been traditionally recognized for its relevance to bone and liver metabolism, and recent studies have suggested that it is also associated with cardiovascular diseases and atherosclerosis (16, 28). This study identified a positive correlation between ALP and postoperative stroke in patients with MMD undergoing surgical treatment with univariate and multivariate Cox model analyses, suggesting it as a potential indicator of vascular-related prognoses. In particular, a nomogram was constructed to reflect the indicators that may affect the postoperative outcomes of patients with MMD, and is feasible for clinical use.

Recent clinical studies have elucidated the level of ALP is associated with vascular calcification, metabolic syndrome and the increased incidence of mortality (16). It was thought to through mechanisms such as reflecting inflammation, endothelial dysfunction and affecting vascular homeostasis (23). Recent study have also shown that serum ALP was associated with cerebral small vessel disease and impaired cerebral microcirculation (29). Besides, ALP played a crucial role in promoting angiogenesis during the fracture healing process, and ALP and VEGF exhibit a complex and coordinated relationship in promoting osteogenesis and angiogenesis (30). Hence, ALP may contribute to the pathogenesis of MMD. Emerging evidence in cardiovascular research has revealed that ALP may play a broader and more complex role than its traditional functions. Elevated ALP levels have been implicated in vascular calcification and atherosclerosis, suggesting a link between ALP activity and cardiovascular diseases (31, 32). In the context of atherosclerotic disease, ALP contributes to the ossification of vascular smooth muscle cells, leading to arterial stiffening and potential plaque instability (33, 34). Similar enzymatic processes may be relevant in the cerebral vasculature of patients with MMD, where fragile collateral networks are inherently prone to disturbances in hemodynamic forces. The association between ALP and vascular inflammation, calcification, and endothelial dysfunction suggests its involvement in pathways that could exacerbate cerebrovascular risk, particularly following surgical treatment (35, 36).

Our findings align with and extend previous clinical observations demonstrating the prognostic value of serum ALP levels in cerebrovascular diseases. Multiple cohort studies have established that elevated ALP levels are independently associated with an increased risk of incident stroke, with meta-analyses showing a dose-response relationship between ALP levels and stroke incidence across diverse populations (37). Importantly, our results corroborate emerging evidence that ALP elevation predicts poor post-stroke outcomes, including higher risks of stroke recurrence, mortality, and functional disability (19, 21, 22). The consistency between our findings in MMD patients and these previous observations in general stroke populations suggests that ALP may represent a biomarker in MMD patients.

Emerging evidence suggests that ALP contributes to cerebrovascular pathology by mediating vascular calcification, inflammatory responses, and endothelial dysfunction. The dual role of ALP in vascular biology—promoting both pathological calcification and compensatory angiogenesis through VEGF interaction—creates a complex dynamic in MMD progression and postoperative recovery (30, 32). While ALP-mediated angiogenesis may support collateral circulation formation, excessive ALP activity appears to dominate in the postoperative period, potentially leading to graft dysfunction and recurrent ischemic events. Mechanistically, ALP may disrupt inorganic pyrophosphate (PPi) metabolism—a potent endogenous inhibitor of vascular calcification—thereby promoting calcium deposition in cerebral vessels and exacerbating arteriosclerosis and ischemic events (32, 38). These pathological processes may also explain why elevated ALP levels correlate with cerebral small vessel disease and post-bypass cerebrovascular complications, suggesting its potential as both a pathogenic factor and prognostic biomarker in MMD.

Vascular calcification may be a key factor for cerebrovascular events (39). ALP plays a critical role in the metabolism of organic pyrophosphate, a key inhibitor of vascular calcification (32). Organic pyrophosphate acts as a physiological regulator, preventing the excessive deposition of calcium and phosphate in the vascular walls (32). Cerebral vascular calcification contributes to the pathogenesis of arteriosclerosis, which subsequently leads to cerebral ischemic events. In addition, ALP was associated with cerebrovascular events after stroke and cerebral artery stenosis bypass grafting surgery (19, 23). Hence, increasing ALP levels may be a risk factor or a new biomarker for cerebrovascular events after MMD bypass surgery.

Previous studies have demonstrated that RNF213 was associated with the progression of MMD, but no significant correlation has been found with either long-term or short-term prognosis. Therefore, it was not included as a variable in the construction of the nomogram. The nomogram offers key statistical and practical advantages. First, it integrates multiple predictors into a single, easy-to-use visualization, converting complex statistical models into an intuitive tool. Second, it provides individualized, quantitative risk predictions—offering absolute probabilities rather than only relative risks, thereby supporting more precise clinical decisions. Finally, it retains more predictive information compared to categorical staging systems and is more accessible than digital calculators, making it both accurate and practical for clinical use.

Although our study provides novel insights into the role of ALP in MMD, there are still limitations that require further research. As a prospective study, long-term follow-up data and more comprehensive test results are required, and. Furthermore, the homogeneity of our sample, stemming from a single center, imposes restrictions on generalizability. Prospective multicenter studies with larger and more diverse populations are required. Despite our efforts to mitigate confounding through multivariate adjustment and model selection, the potential for residual confounding remains. Although we accounted for major clinical and demographic variables, unmeasured or unknown factors not included in the analysis may still influence the outcomes, which unmeasured confounding factors could distort the true association between serum ALP and post-operative stroke, complicating causal inference. Finally, the dichotomization of ALP levels may represent a significant limitation, as it fails to capture the prognostic continuum within the normal range. A more granular analysis of ALP gradients is needed to validate and extend our findings. Future studies with more comprehensive data collection are warranted to further validate these findings.

## Conclusion

This study demonstrated that serum ALP could be a potential predictive marker for postoperative stroke events in patients with MMD. It may be helpful to construct a more integrative approach to perioperative assessment, encompassing biochemical and clinical data, to optimize risk prediction. Further investigations are necessary to elucidate the pathophysiological mechanisms underlying this association. ALP, a modifiable risk factor, may be a tailored strategy for managing patients with MMD in the perioperative period.

## Data Availability

The datasets presented in this article are not readily available because the datasets used and/or analyzed during the current study are available from the corresponding author upon reasonable request. Requests to access the datasets should be directed to Peicong Ge, gepeicong@163.com.

## Supplementary material

Supplementary Figure S1 The calibration curve of the nomogram.

Supplementary Figure S2 The RCS of between ALP concentration and all postoperative events. **(A)** The crude model was concerned the unadjusted model of serum ALP concentrations. **(B)** The final model was adjusted for age, gender, SBP, DBP, BMI, ALP and HGB concentrations. MMD, moyamoya disease; SBP, systolic blood pressure; DBP, diastolic blood pressure; BMI, body mass index; WBC, white blood cell; RBC, red blood cell; HGB, hemoglobin; PLT, platelet; AST, aspartate aminotransferase; ALT, alanine transaminase; ALP, alkaline phosphatase.
