# Supplementary figures and images for: Alkaline phosphatase predicts short-term postoperative outcome in adult patients with moyamoya disease
Source: Front Neurol. 2025 Sep 26;16:1606348. doi: 10.3389/fneur.2025.1606348 (PMC12510858; doi:10.3389/fneur.2025.1606348)

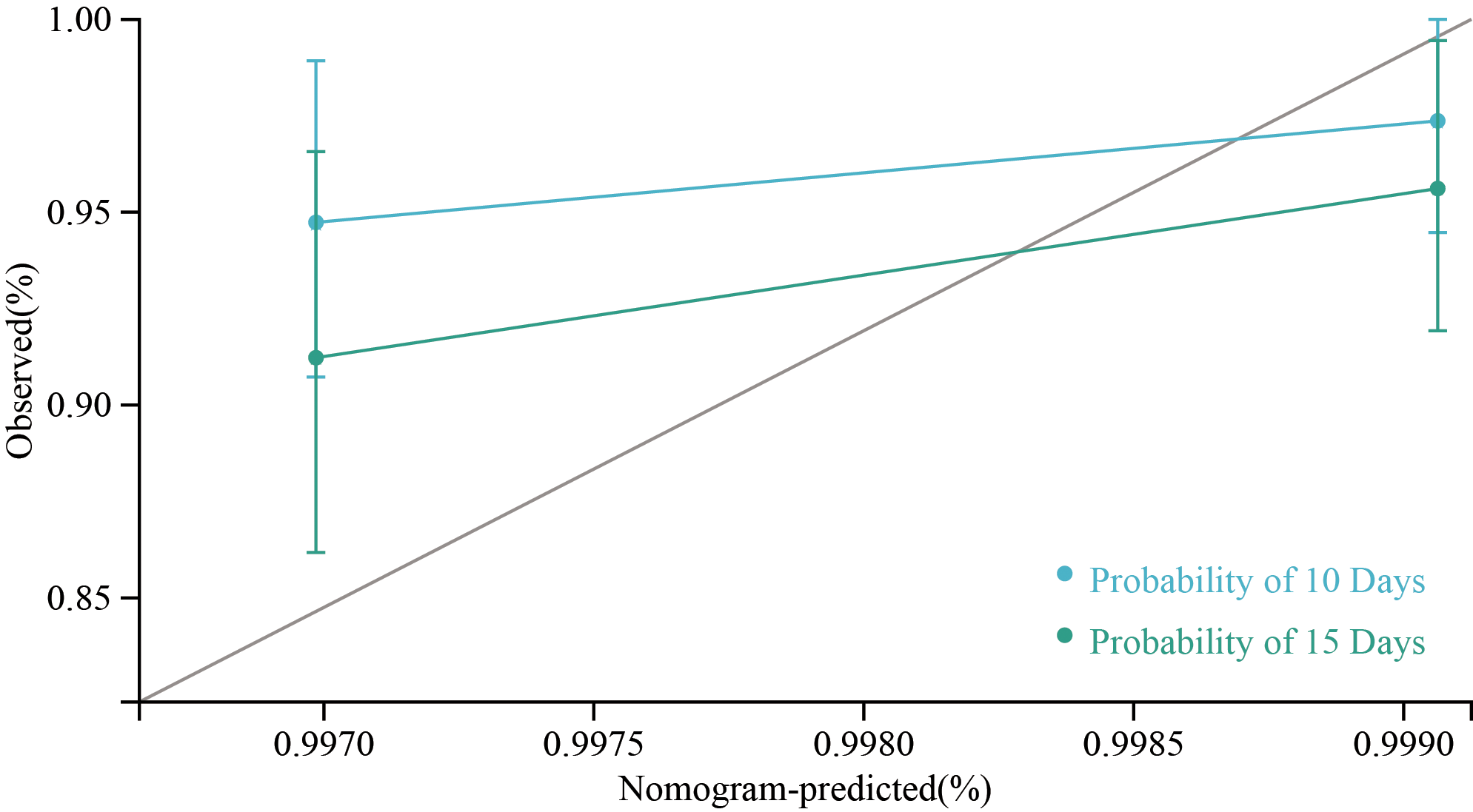

Supplement: Supplementary Figure S1 — The calibration curve of the nomogram. [file Image_1.tif]

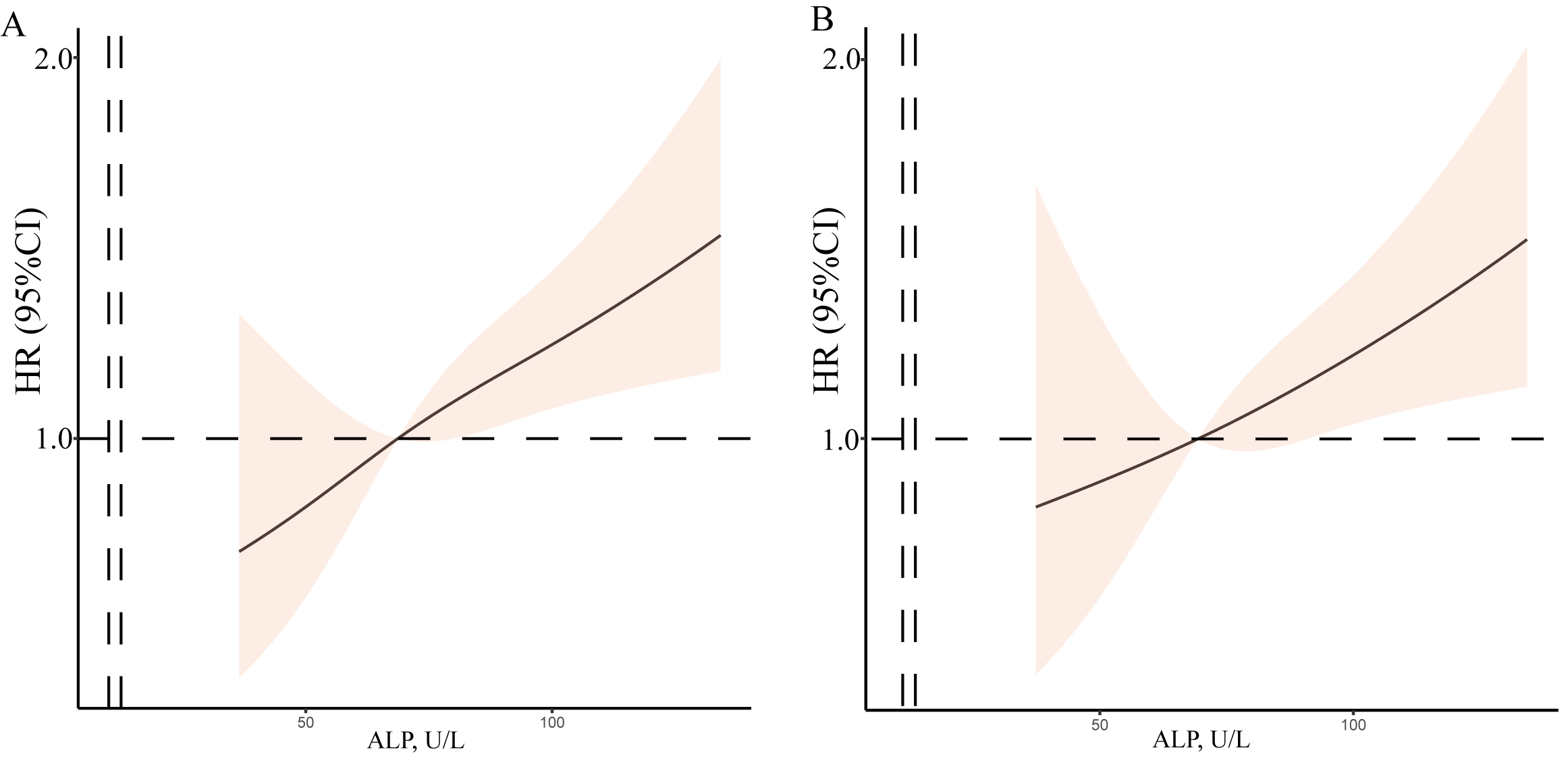

Supplement: Supplementary Figure S2 — The RCS of between ALP concentration and all postoperative events. (A) The crude model was concerned the unadjusted model of serum ALP concentrations. (B) The final model was adjusted for age, gender, SBP, DBP, BMI, ALP and HGB concentrations. MMD, moyamoya disease; SBP, systolic blood pressure; DBP, diastolic blood pressure; BMI, body mass index; WBC, white blood cell; RBC, red blood cell; HGB, hemoglobin; PLT, platelet; AST, aspartate aminotransferase; ALT, alanine transaminase; ALP, alkaline phosphatase. [file Image_2.tif]
